# EMMPRIN expression positively correlates with WHO grades of astrocytomas and meningiomas

**DOI:** 10.1007/s11060-013-1184-5

**Published:** 2013-07-02

**Authors:** Wen-Chiuan Tsai, Ying Chen, Li-Chun Huang, Herng-Sheng Lee, Hsin-I Ma, Shih-Ming Huang, Huey-Kang Sytwu, Dueng-Yuan Hueng

**Affiliations:** 1 Department of Neurological Surgery, Tri-Service General Hospital, National Defense Medical Center, 325, Sec. 2, Cheng-Kung Road, Neihu, Taipei, 114 Taiwan, ROC; 2 Department of Pathology, Tri-Service General Hospital, National Defense Medical Center, Taipei, Taiwan, ROC; 3 Department of Biology and Anatomy, National Defense Medical Center, Taipei, Taiwan, ROC; 4 Department of Microbiology and Immunology, National Defense Medical Center, Taipei, Taiwan, ROC; 5 Department of Biochemistry, National Defense Medical Center, Taipei, Taiwan, ROC

**Keywords:** Astrocytoma, Meningioma, EMMPRIN, GEO profile, WHO grades, Tumor stem-like cells

## Abstract

High-grade primary brain tumors possessed poor outcome due to invasiveness. Extracellular matrix metalloproteinase inducer (EMMPRIN) stimulates peri-tumoral fibroblasts to secrete matrix metalloproteinase and promote invasiveness. This study hypothesized that high-grade brain tumors overexpress EMMPRIN. Analyzing the public delinked database from the Gene Expression Omnibus profile, the results showed that the EMMPRIN mRNA level was higher in WHO grade IV (*n* = 81) than in grade III (*n* = 19, *p* < 0.0005) astrocytomas and non-tumor brain tissue controls (*n* = 23, *p* < 0.00001). The results of tissue microarray-based immunohistochemical (IHC) staining revealed that EMMPRIN levels positively correlated with WHO grades for astrocytomas (*p* = 0.008) and meningiomas (*p* = 0.048). EMMPRIN mRNA levels in conventional glioma cell lines (*n* = 36) was not less than those in glioma primary culture cells (*n* = 27) and glioblastoma stem-like cells (*n* = 12). The GBM8401, U87MG, and LN229 human glioma cell lines also overexpressed EMMPRIN. Hematoxylin and eosin, IHC, and immunofluorescence staining of xenografts confirmed that high-grade brain tumors overexpressed EMMPRIN. Lastly, Kaplan–Meier analysis revealed poorer survival in WHO grade IV (*n* = 56) than in grade III astrocytomas (*n* = 21, by log-rank test; *p* = 0.0001, 95 % CI: 1.842–3.053). However, in high-grade astrocytomas, there was no difference in survival between high and low EMMPRIN mRNA levels. Thus, this study identified that high-grade brain tumors overexpress EMMPRIN, which positively correlates with WHO grades in human astrocytomas and meningiomas, and suggests that EMMPRIN may be a therapeutic target of brain tumor.

## Introduction

Astrocytomas and meningiomas are the two most common primary brain tumors (PBTs) [1]. Proper histologic grading is a major predictor of therapeutic effect and prognosis in PBTs [2]. However, the discrepancy between histologic classification and aggressive behavior in meningiomas is around 7–20 % [3]. Therefore, the investigation of biomarkers is very important to bridge the gap. The Gene Expression Omnibus (GEO) profiles [4, 5] provided public delinked database for high-throughput investigation of biomarkers.

Matrix metalloproteinases degrade all of the molecules of the extracellular matrix (ECM) [6]. Activation of MMPs greatly affects cell–matrix interaction, angiogenesis, and the inflammatory process [7–9]. Extracellular MMP inducer (EMMPRIN), also called CD147 or basigin (BSG), is a 55-kDa molecule expressed on the tumor cell surface that plays important roles in the up-regulation of peri-tumoral fibroblasts [10]. EMMPRIN is also an inducer of MMP-1, MMP-2, and MMP-3 [6, 11]. Recent studies have shown that ECM degradation may be related to tumor invasion, progression, and even metastasis [12]. Thus, EMMPRIN overexpression is seen in many human cancers, including oral [13], laryngeal [14], esophageal [15], breast [16], ovarian [17], cervical [18], hepatic [19], kidney [20], colorectal carcinomas [21], and T cell lymphomas [22]. EMMPRIN on the surface of glioma cells may also strengthen tumor progression [23, 24], but the possible role of EMMPRIN in meningiomas remains unknown.

The present study hypothesized that high-grade brain tumors overexpress EMMPRIN. Using the GEO profiles, EMMPRIN expressions in PBTs were investigated and correlated with World Health Organization (WHO) grades and survival. Immunohistochemical (IHC) staining of tissue microarray and xenografts were used to validate the database findings. This study posited that a simple strategy of combined bioinformatics and wet lab approaches can be used to identify potential biomarkers in human brain tumors.

## Materials and methods

### Analyses of functional genomic databases

The EMMPRIN mRNA expression was obtained from the GDS1962/208677_s_at/BSG database. http://www.ncbi.nlm.nih.gov/geo/tools/profileGraph.cgi?ID=GDS1962:208677_s_at. The sample size and WHO grades enrolled in this GDS1962 dataset was described previously [25]. The survival data of 77 primary high-grade astrocytomas (WHO grades III and IV) obtained from the GDS1815 database (http://www.ncbi.nlm.nih.gov/geo/tools/profileGraph.cgi?ID=GDS1815:208677_s_at) and devoted by Phillips et al. [26]. The GDS3885 database included 36 panels of conventional glioma cell lines data, 12 panels of glioma primary culture cells data, and 27 panels of glioma stem-like cells data (http://www.ncbi.nlm.nih.gov/geo/tools/profileGraph.cgi?ID=GDS3885:208677_s_at). The background of cell line was described previously [27].

### Construction of tissue microarray

The Institutional Review Board of Tri-Service General Hospital, National Defense Medical Center approved this study. Tissue microarray slides were constructed from 103 paraffin-embedded tissues, including 59 cases of meningiomas, 41 cases of astrocytomas, and 3 non-neoplastic brain tissues. For slide construction, one core (2 mm in diameter) was taken from a selected area of each paraffin-embedded tumor tissue. Both the tissue microarray scores and the corresponding paraffin-embedded specimens were uniformly stained. Pathologic diagnosis in these cases was reviewed by at least two experienced pathologists. The histo-pathologic differentiation of brain tumors was determined according to the criteria of the WHO classification of tumor [28]. None of the astrocytomas and meningiomas included in this study ever received radiotherapy or chemotherapy before surgery.

### Immunohistochemical (IHC) staining

Tissue microarray sections and xenografts were processed following previous protocol [2], incubated with a polyclonal rabbit anti-human EMMPRIN antibody (1:200 diluted in phosphate buffered saline [PBS]; Bioss, Woburn, MA, USA) for 1 h at room temperature, washed 3 times (each for 5 min in PBS), incubated with biotin-labeled secondary immunoglobulin (1:100, DAKO, Glostrup, Denmark) for 1 h at room temperature, washed 3 times, and treated with 3-amino-9-ethylcarbazole substrate chromogen (DAKO, Glostrup, Denmark) at room temperature to visualize peroxidase activity. Sections of poorly differentiated hepatocellular carcinoma tissue (known to stain positive for EMMPRIN) were used as positive control and non-neoplastic liver were used as negative control [19].

### Assessment of EMMPRIN immuno-staining scores

To evaluate immuno-reactivity and histologic appearance, all tissue microarray experiments were repeated two times and the slides were examined and scored by two investigators concurrently. To assess EMMPRIN immuno-staining scores, the intensity of cytoplasmic and membranous staining was scored as 0 (absence of staining), 1 (weak staining), 2 (moderate staining), or 3 (strong staining). Weak, moderate, and strong cytoplasmic stainings were identified by microscopy with magnification of 40×, 20×, and 10× or 4×, respectively [29]. The cut-off value of EMMPRIN intensity scores was set by the magnification of objective lens. The percentage of tumor staining was scored 0 (<5 %), 1 (5–25 %), 2 (25–50 %), 3 (50–75 %), and 4 (75–100 %). The EMMPRIN immuno-staining scores of human astrocytomas and meningiomas were defined by calculating the intensity score and multiplying it by the corresponding percentage score. EMMPRIN immuno-staining scores ranged from 0 to 12, including 0, 1, 2, 3, 4, 6, 8, 9, and 12.

### Cell lysate preparation and Western blots

GBM8401, U87MG, LN229, and HepG2 were maintained in Dulbecco’s modified Eagle’s medium (DMEM) containing 10 % fetal bovine serum (FBS), penicillin, and streptomycin. Cell lysates prepared from 2 × 10^7^ GBM8401, U87MG, LN229 glioma cells, and HepG2 hepatoma cell line (positive control) were used for Western blot analysis of EMMPRIN, with α-actin (Santa Cruz) as internal control following previous protocol [30].

### In vivo orthotopic xenograft

The Institutional Animal Care and Use Committee of National Defense Medical Center, Taipei, Taiwan (IACUC-13-147) approved the study of xenografts. The experimental procedures of in vivo orthotopic xenograft were as described previously [25, 30–32]. Briefly, 5 × 10^5^ cells were injected into the right cerebral hemisphere of BALB/cAnN.Cg-Foxn1nu/CrlNarl nude mice. Gliomas were permitted to grow in the murine brain.

### Immunofluorescence (IF) staining

After deparaffinization and rehydration, the tumor samples were rinsed with PBS. For antigen retrieval, the samples were heated by a microwave oven in citrate buffer (10 mM Citric Acid, pH 6.0) for 15 min. After cooling for 15 min, blocking solution (5 % non-fat milk in 0.1 % Triton X-100) was applied to block non-specific binding at room temperature for 30 min. Then, primary antibody (EMMPRIN) dissolved in PBS with 5 % non-fat milk was added to the samples at 4 °C overnight. The next day, the samples were rinsed with PBS and secondary antibody-conjugated FITC and DAPI (Sigma) were applied at room temperature for 1 h and 15 min, respectively. The samples were mounted (Gel mount Aqueous, Sigma) and the slides were examined by microscopy and photographed (Leica).

### Statistical analysis

All results were expressed as mean ± standard deviation (SD).The correlations between variables of immuno-staining scores were calculated by the Pearson Product Moment Method. Student’s two-tailed t test was used to determine differences in means between any two groups in EMMPRIN mRNA levels of different WHO grades of human astrocytoma samples. SigmaState software (Jandel Scientific, USA) was also used to perform linear regression testing to analyze the relationships between EMMPRIN expression and WHO grades of astrocytomas and meningiomas. Survival cures were analyzed using the Kaplan–Meier method, while differences in survival distributions were investigated by log-rank test. Statistical significance was set at *p* < 0.05.

## Results

### GEO identified the candidate biomarker EMMPRIN mRNA level and correlated it with WHO grades in human astrocytomas

**Fig. 1:**
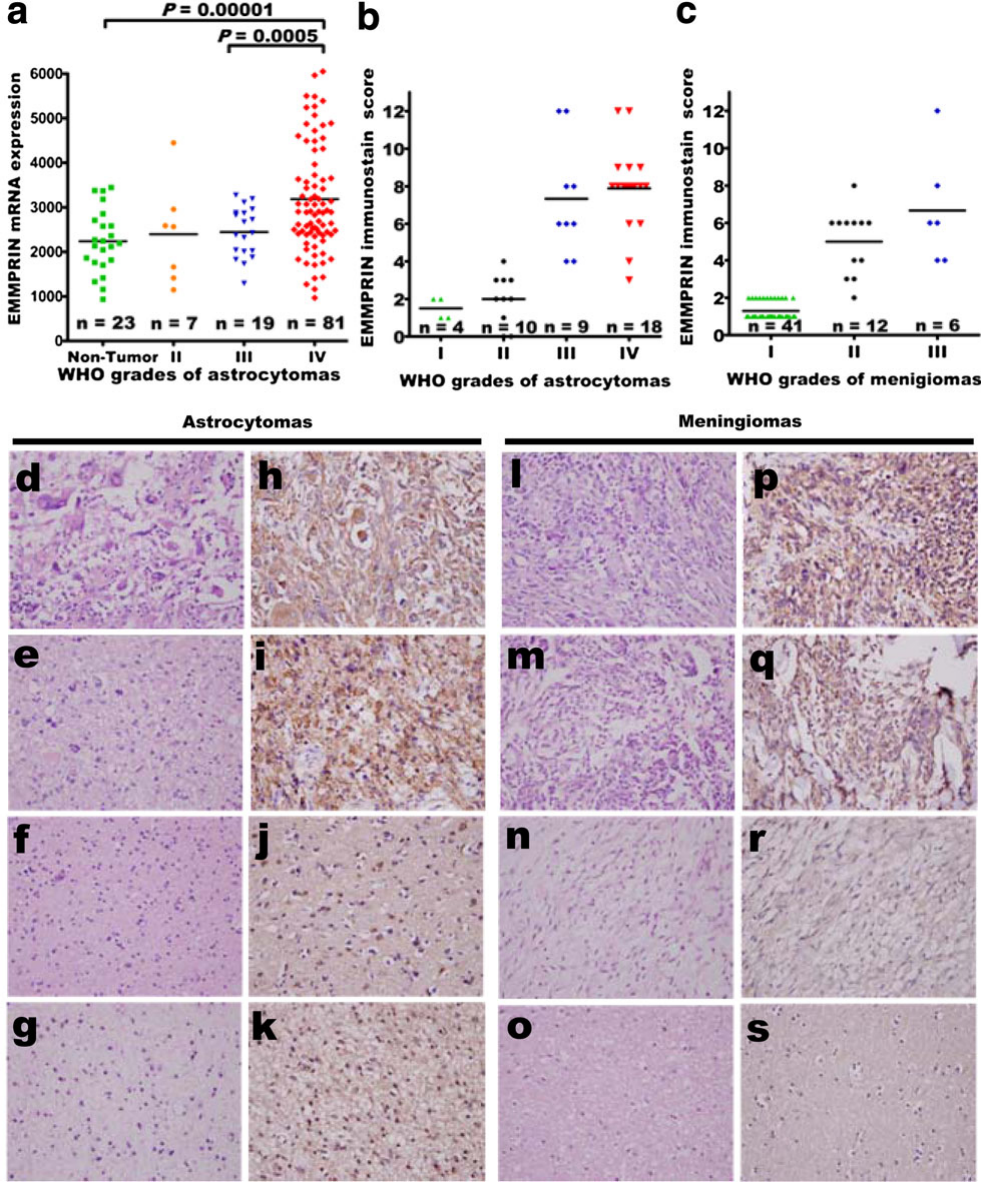
Expression of EMMPRIN in human astrocytomas and meningiomas. **a** EMMPRIN mRNA levels were significantly higher in grade IV than grade III (*p* = 0.0005) primary astrocytomas patient samples and non-tumor controls (*p* = 0.00001). **b**, **c** By immunostain score, EMMPRIN positively correlated with WHO grades in human astrocytomas (*p* = 0.008), and meningiomas (*p* = 0.048, by Pearson product method correlation test), respectively. Representative hematoxylin and eosin staining of **d** glioblastomas multiforme, **e** anaplastic astrocytomas, **f** diffuse astrocytomas, and **g** pilocytic astrocytomas. Immuno-histochemical staining of EMMPRIN in **h** glioblastomas multiforme, **i** anaplastic astrocytomas, **j** diffuse astrocytomas, and **k** pilocytic astrocytomas. Representative hematoxylin and eosin staining of **l** anaplastic, **m** atypical, **n** meningothelial meningiomas, and **o** non-neoplastic brain tissues. Immuno-histochemical staining of EMMPRIN in **p** anaplastic, **q** atypical, **r** meningothelial meningiomas, and **s** non-neoplastic brain tissues. Original magnification ×400

The GDS1962 dataset and statistical analyses were used to investigate the correlation between EMMPRIN mRNA and WHO grades (Fig. 1a). In samples with different pathologic grades, EMMPRIN mRNA level was higher in WHO grade IV (*n* = 81) than in grade III (*n* = 19) (*p* < 0.0005) human astrocytomas and non-tumor brain tissue controls (*n* = 23) (*p* < 0.00001, Student’s *t* test).

### Tissue microarray provided the platform to identify EMMPRIN and correlate with WHO grades in human astrocytomas

Only weak stain and limited percentage of non-neoplastic brain tissues were expressed by the IHC staining of EMMPRIN (average immuno-staining scores, 0.5 ± 0.4). Compared to non-neoplastic brain tissues, most astrocytomas had significantly higher expressions of EMMPRIN. Moreover, the IHC staining intensity and average percentage of tumor staining in anaplastic astrocytomas (average score, 7.3 ± 3.0) and glioblastoma multiforme (average score, 7.8 ± 2.1) were higher than in pilocytic astrocytomas (average score, 1.5 ± 0.5) and diffuse astrocytomas (average score, 2.0 ± 1.3). Increased EMMPRIN expression positively correlated with WHO grade of astrocytomas (*p* = 0.008) (Fig. 1a, b, d–k; Table 1).

**Table 1:** The immunohistochemical staining scores of EMMPRIN in various WHO grades of astrocytomas and meningiomas

	Case numbers	Average intensity score (means ± standard error)	Average percentage score (means ± standard error)	Average immunostain score (means ± standard error)	Correlation^a^
Non-neoplastic brain tissue^b^	3	0.3 ± 0.2	0.5 ± 0.3	0.5 ± 0.4	
WHO grades of astrocytomas					
Pilocytic astrocytoma, WHO grade I	4	0.8 ± 0.3	1.5 ± 0.6	1.5 ± 0.5	Positive correlation (*p* = 0.008*)
Diffuse astrocytoma, WHO grade II	10	1.1 ± 0.8	1.4 ± 0.5	2.0 ± 1.3	
Anaplastic astrocytomas, WHO grade III	9	2.6 ± 1.3	2.8 ± 1.1	7.3 ± 3.0	
Glioblastoma multiforme WHO grade IV	18	2.5 ± 1.2	3.1 ± 0.9	7.8 ± 2.1	
WHO grades of meningiomas					
Meningiomas, WHO grade I	41	0.8 ± 0.4	1.2 ± 0.5	1.3 ± 0.6	Positive correlation (*p* = 0.048*)
Atypical meningiomas, WHO grade II	12	1.8 ± 0.8	2.9 ± 1.2	5.0 ± 1.8	
Anaplastic meningiomas, WHO grade III	6	2.1 ± 1.4	3.1 ± 1.4	6.6 ± 3.1	

*EMMPRIN* Extracellular matrix metalloproteinase inducer, *WHO* World Health Organization

* Means statistical significance

^a^The correlation was analyzed by Pearson Product Method Correlation test

^b^These three non-neoplastic brain tissues were taken from non-tumor part of diffuse astrocytomas (WHO grade II)

### Tissue microarray provided the platform to identify EMMPRIN and correlate with WHO grades in human meningiomas

The IHC staining intensity, percentage of meningioma expression, and total immuno-staining scores of EMMPRIN were shown in Table 1. Most meningiomas showed higher EMMPRIN immuno-staining scores compared to non-tumor brain tissues. Furthermore, the intensity and immuno-staining scores of EMMPRIN in atypical meningiomas (5.0 ± 1.8) and anaplastic meningiomas (6.6 ± 3.1) were higher than that of meningothelial meningiomas (1.3 ± 0.6). The immuno-staining scores of EMMPRIN significantly correlated with WHO grades of meningiomas (*p* = 0.048) (Fig. 1c, l–s; Table 1).

### In vitro validation using GEO bioinformatics analysis of the EMMPRIN mRNA expression in glioma cell lines

**Fig. 2:**
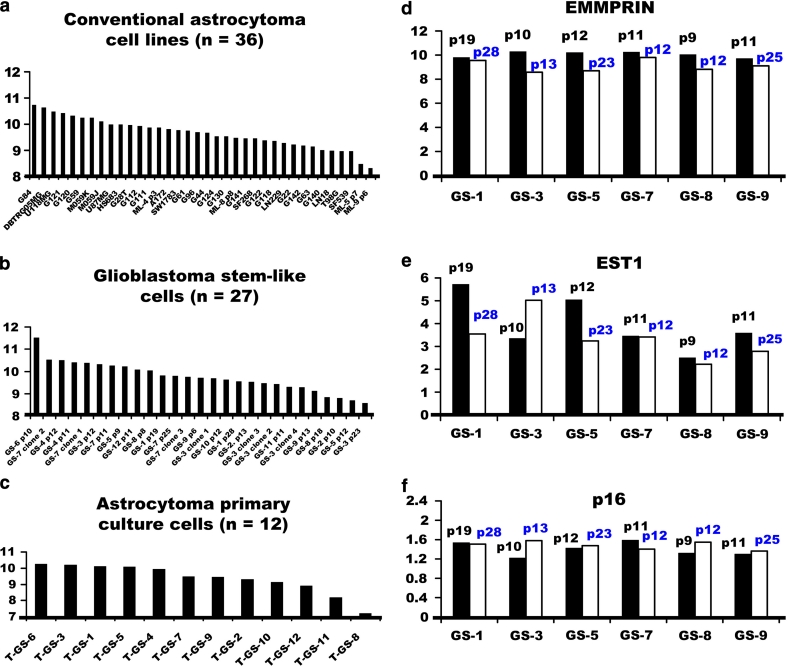
Comparison of the EMMPRIN mRNA expressions among **a** conventional astrocytoma cell lines (*n* = 36), **b** glioblastoma stem-like cells (*n* = 27), and **c** astrocytoma primary culture cells (*n* = 12) from GDS3885 dataset of GEO profile. Glioma cell lines recapitulated the transcriptional value of EMMPRIN in the above three types of glioma cells. **d**–**f** Alterations of EMMPRIN, EST1, and p16 mRNA expressions after serial passages. The mRNA expression in serial passages of glioma-stem like cells showed decreased EMMPRIN and EST1, but increased p16 when comparing early to late passages

Using the GDS3885 database, the EMMPRIN mRNA expression in conventional glioma cell lines, glioma primary culture cells, and glioma stem-like cells were analyzed. There was no significant difference among conventional glioma cell lines (*n* = 36), glioma primary culture cells (*n* = 27), and glioblastoma stem-like cells (*n* = 12) from human primary brain gliomas (Fig. 2a–c). This suggests that glioma cell lines recapitulate transcriptional features of gliomas, thereby allowing the investigation of therapeutic candidates.

### Serial passages on alterations of EMMPRIN, EST1, and p16 mRNA expression profiles

Regarding the GDS3885 database, eight pairs of serial passages data were achieved from the early and late passages of glioma stem-like cells. EMMPRIN mRNA expression decreased in six of the eight pairs (75 %), including GS-1, GS-3, GS-5, GS-7, GS-8, and GS-9 when comparing the early with the late passages (Fig. 2d). Since serial passage decreased the amount and length of telomeric DNA in human fibroblasts [33], the mRNA expression of telomere-regulated genes was further checked. Ever shorter telomeres protein 1 (EST1) [34] was one of the telomere-regulated sub-units directly involved in telomere replication and correlated with telomerase. Analyses of EST1 showed higher EST1 in early passage than in late passage (Fig. 2e). Since the reduction of telomere length led to senescence, the p16 belonged to the senescence-regulated gene [35]. Further investigation of the mRNA expression of p16 revealed that later passage expressed higher level of p16 than early passage in four of six glioma stem-like cells, including GS-3, GS-5, GS-8, and GS-9 (Fig. 2f), suggesting the serial passage-induced senescence of gliomas.

### In vitro validation using Western blot analysis confirmed the overexpression of EMMPRIN in glioma cells

**Fig. 3:**
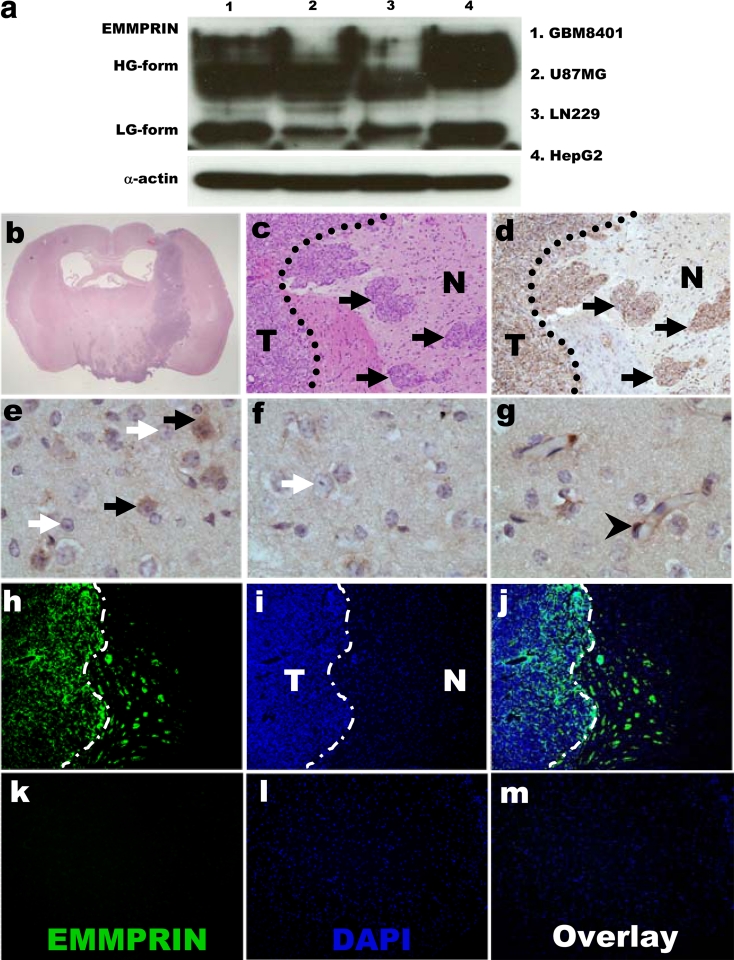
Western blot analysis of astrocytomas cell lines, and histopathologic staining of EMMPRIN-expressing in GBM8401 glioma xenografts. **a** Western blot analysis of EMMPRIN expression in GBM8401, U87MG, LN229 glioma cells, and positive control HepG2 hepatoma cell line. EMMPRIN protein production consisted of *HG* high glycosylation, and *LG* low glycosylation forms. The expression of α-actin was used as internal control. Hematoxylin and eosin staining of GBM8401 glioma xenograft in **b** coronal section of the brain and **c** tumor–brain interface. **d** Immuno-histochemical staining revealed EMMPRIN-expressing invasive tumor islands (*black* *arrows*). **e**–**g** The normal control of astrocytes (*black arrows*), oligodendrocytes (*white arrows*), and endothelial cells (*black* *arrow* *head*). In the immuno-fluorescence staining with antibodies specific for EMMPRIN, **h** FITC-labeled EMMPRIN-expressing GBM8401 glioma cells were distributed near the invasive front at the tumor–brain interface (*dash line*) with some invasive tumor islands away from the main tumor mass. **k** The contralateral normal control of brain astrocytes showed only faint FITC fluorescence. **i**, **l** DAPI-stained nuclei. **j**, **m** The merged photos of FITC-labeled EMMPRIN expressing tumors and DAPI-stained nuclei. Original magnification ×100. Data were representative of three independent experiments. *T* tumor, *N* neighborhood brain parenchyma. Original magnification ×12.5 (**b**); ×200 (**c**, **d**); ×1,000 in (**e**–**g**); and ×100 (**h**–**m**). (Color figure online)

To determine the EMMPRIN protein production in human glioma cell line, Western blots showed that EMMPRIN protein production was overexpressed in human glioma cell lines (Fig. 3a) and was higher in GBM8401 than in U87MG, or LN229 glioma cell lines. The high EMMPRIN-expressing GBM8401 was then used for further xenograft study.

### Immunohistochemical and immunofluorescence staining of xenograft confirmed EMMPRIN overexpression in high-grade astrocytomas

The GBM8401-xenograft formed glioma mass in the right hemisphere, with invasive glioma tumor islands (Fig. 3b, c) after 2-week inoculation. The high EMMPRIN-expressing glioma cells also displayed a highly invasive phenotype, with invasive glioma islands growing away from the original xenograft site (Fig. 3d). In contrast, the normal control of astrocytes and endothelial cells showed low-EMMPRIN expression, while oligodendrocytes were negative for EMMPRIN staining (Fig. 3e–g). The high EMMPRIN-expressing glioma cells were further identified via IF staining with antibodies specific for EMMPRIN. The FITC-labeled EMMPRIN-expressing GBM8401 glioma cells were distributed near the invasive front at the tumor–brain interface with some invasive tumor islands away from the glioma main tumor mass, compared to the contralateral normal control of brain astrocytes that showed only faint FITC fluorescence (Fig. 3h–m). Based on these evidences, high-grade brain tumors overexpressed EMMPRIN.

### Relationships of EMMPRIN mRNA to survival of high-grade astrocytomas

**Fig. 4:**
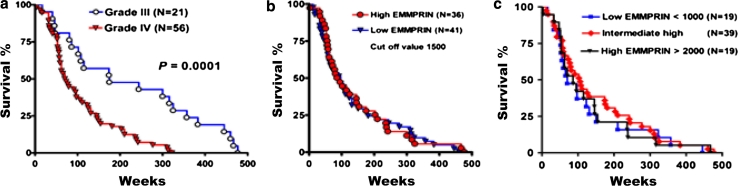
Kaplan-Meier survival analyses of high grade astrocytomas. **a** Survival patterns in patients with WHO grade IV (*n* = 56) compared to those with WHO grade III (*n* = 21) high-grade astrocytomas (*p* = 0.0001, by log-rank test). **b** Patterns of patients with high EMMPRIN (>1,500, *n* = 36) versus low EMMPRIN (<1,500, *n* = 41) expressions (*p* = 0.7274, by log-rank test). **c** Patterns of patients with high EMMPRIN (>2,000, *n* = 19) versus intermediate high (*n* = 39) and low EMMPRIN (<1,000, *n* = 19) expressions (*p* = 0.6981, by log-rank test)

Using the GDS1962 database, survival analysis was achieved from 77 public delinked databases. The median survival time in patients with WHO grade III and grade IV astrocytomas was 175 and 71.5 weeks, respectively. Kaplan–Meier post-operative survival analysis was applied to investigate the relationship between overall survival of patients with high grade astrocytomas and EMMPRIN mRNA levels. Overall survival was poor in patients with WHO grade IV (*n* = 56) than WHO grade III astrocytomas (*n* = 21) (*p* = 0.0001, by log-rank test; 95 % CI: 1.842–3.053) (Fig. 4a). This result consistently indicated that the GDS1962 database was a reliable positive control for survival analysis.

The cut-off value was then set up at 1,500, or the point of median EMMPRIN gene expression value. The median survival time of patients with low and high EMMPRIN expressions was 97 and 90.5 weeks, respectively. Patients with high-grade astrocytomas and high EMMPRIN mRNA levels (*n* = 36) did not have worse overall survival than patients with low EMMPRIN mRNA expression (*n* = 41) (*p* = 0.7274, by log-rank test; 95 % CI: 0.4327–1.711) (Fig. 4b). Kaplan–Meier post-operative survival analysis did not show any statistical significance when the cut-off value of EMMPRIN expression was set to low (<1,000, *n* = 19), intermediate (1,000–2,000, *n* = 39), and high (>2,000, *n* = 19) (Fig. 4c).

## Discussion

This study validates a simple strategy using GEO bioinformatics and wet lab approaches to identify the biomarker, EMMPRIN, in human brain tumors. Li et al. [36] investigated EMMPRIN (also known as CD147) mRNA expression and IHC in 50 cases and found that EMMPRIN expression levels were apparently elevated in high-grade astrocytomas. This was consistent with the finding of the present study. Their study also found that higher expression of EMMPRIN was associated with poor survival time in their astrocytoma patient cohort. In contrast, regarding the stratification of EMMPRIN gene expression level, Kaplan–Meier survival analysis of the GDS1815 database showed no statistical difference in 77 primary high grade astrocytomas, suggesting that EMMPRIN was not the only factor that played a role in determining outcome. Other factors, such as nodal and osteopontin, are also important contributors to invasiveness and survival in astrocytomas [25, 30, 32]. The other possible explanations include the limitation of patient populations that only involved high-grade, but not low-grade, controls.

Complete resections of meningiomas and tumor bed meninges have the advantage of reducing recurrence in benign or atypical meningiomas, but the results are not satisfactory [37]. This suggests that there are still other molecular factors like EMMPRIN and MMPs aiding tumor cell invasion to move away from the main tumor mass and infiltrate the normal brain parenchyma, leading to tumor recurrence.

The paradigm of tumor stem-like cells has been established in human astrocytomas [31] and meningiomas [38–41]. Human glioma cells exhibit radio-resistance and higher colony formation ability in CD133-expressed glioma sub-populations [31]. Consistently, human meningioma stem-like cells display chemotherapeutic resistance, radiation resistance, high tumorigenicity, and infiltration into the surrounding normal brain parenchyma [40]. One of the interesting findings is the trend of EMMPRIN mRNA expression decreasing in 75 % of serial passages of glioma stem-like cells. It has been reported that in vitro serial passage decreases the amount and length of telomeric DNA in human fibroblasts [33]. Further analyzing EST1, one of the telomere-regulated sub-units, showed that early passage display higher EST1 than late passage, consistent with the serial passage effect. Other possible reasons include senescence. Investigation of p16 revealed that later passage expressed higher p16 level than early passage in 75 % of glioma stem-like cells, suggesting the serial passage-induced senescence of gliomas. Overexpression of p16 induces senescence and cell cycle arrest to suppress the invasion and proliferation of human glioma cell lines U87MG and U373 MG. The p16-overexpressed glioma cells are positive for senescence-associated beta-galactosidase staining and enlarged morphology [35].

It is difficult to obtain the hundred number scales of fresh or frozen samples from human brain tumors, especially for low-grade astrocytomas, to analyze the mRNA expression or protein level. Instead, using the GEO profile provides an enormous public delinked database for the investigation of molecular cell biology of human brain astrocytomas [5, 25]. However, there are still some limitations inherent to this study. One database alone cannot provide all the information at the same time for investigating mRNA gene expression, protein level, survival time, and clinico-pathologic parameters. To overcome this, multiple databases, including GDS1962, GDS3885, and GDS1815, were used. In meningiomas, limited by the tissue sample amount and GEO database source, the mRNA expression, survival, and cell line data were not obtained in this study. Instead, tissue microarray-based immuno-histochemical staining provided immuno-staining score for study of meningioma samples. Sufficient human meningioma samples in future studies are warranted to allow extensive studies of EMMPRIN.

## Conclusions

This study demonstrates a simple strategy of using GEO bioinformatics and wet lab approaches to identify potential biomarkers in human brain tumors. High-grade brain tumors overexpress EMMPRIN and EMMPRIN expression positively correlates with WHO grades in human astrocytomas and meningiomas, suggesting that EMMPRIN may be a therapeutic target of brain tumor. Future genetic-modified tumor cell manipulation, such as silencing EMMPRIN, may identify the role of EMMPRIN in modulating invasiveness and the underlying signaling pathway.
